# Supplementary material for: Mechanisms governing the pioneering and redistribution capabilities of the non-classical pioneer PU.1
Source: Nat Commun. 2020 Jan 21;11:402. doi: 10.1038/s41467-019-13960-2 (PMC6972792; doi:10.1038/s41467-019-13960-2)
Supplement: Supplementary file 7 — Source data [file 41467_2019_13960_MOESM7_ESM.zip › Source_Data/FigureS2/FigureS2B_MotifScanOutput/KmeansCluster1/homerResults/motif21.similar.html]

motif21

## Information for motif21

A
G
T
C
A
G
C
T
T
C
G
A
A
C
T
G
G
A
T
C
A
G
T
C
C
G
T
A
T
C
A
G
C
G
T
A
A
C
T
G
G
T
A
C
C
G
T
A
  
Reverse Opposite:  

C
G
A
T
A
C
T
G
G
T
A
C
A
C
G
T
A
G
T
C
C
G
A
T
C
T
A
G
C
A
T
G
T
G
A
C
A
G
C
T
C
T
G
A
T
C
A
G
  

|  |  |
| --- | --- |
| p-value: | 1e-40 |
| log p-value: | -9.316e+01 |
| Information Content per bp: | 1.885 |
| Number of Target Sequences with motif | 71.0 |
| Percentage of Target Sequences with motif | 1.39% |
| Number of Background Sequences with motif | 36.6 |
| Percentage of Background Sequences with motif | 0.09% |
| Average Position of motif in Targets | 94.4 +/- 51.8bp |
| Average Position of motif in Background | 105.2 +/- 63.4bp |
| Strand Bias (log2 ratio + to - strand density) | 0.0 |
| Multiplicity (# of sites on avg that occur together) | 1.01 |
| Motif File: | file (matrix) reverse opposite |

### Similar de novo motifs found

|  |  |  |  |  |  |  |  |
| --- | --- | --- | --- | --- | --- | --- | --- |
| Rank | Match Score | Redundant Motif | P-value | log P-value | % of Targets | % of Background | Motif file |
| 1 | 0.965 | G A T C A G T C A G C T C T G A A C T G G A T C A G T C C G T A A T C G C G T A C A T G G T A C C G T A | 1e-40 | -92.761505 | 1.34% | 0.08% | motif file (matrix) |
| 2 | 0.917 | G T A C A C G T C G T A A T C G G A T C A G T C C G T A T C A G C G T A C T A G A G T C | 1e-38 | -87.667696 | 1.55% | 0.15% | motif file (matrix) |
| 3 | 0.876 | A C G T A C T G A G T C A C G T A G T C A C G T A C T G A C T G A G T C | 1e-18 | -42.460726 | 1.14% | 0.22% | motif file (matrix) |
